# Ruptured small pancreaticoduodenal artery aneurysm-clinical features similar to pancreatitis: A case report

**DOI:** 10.1097/MD.0000000000032821

**Published:** 2023-03-03

**Authors:** Ya Nan Yu, Yu Shuang Xu, Pei Nie, Cong Cong Min, Xue Li Ding, Yong Hong Xu, Guo Ping Liu, Tao Mao

**Affiliations:** a Department of Gastroenterology, Affiliated Hospital of Qingdao University, Qingdao, Shandong, China; b Department of Radiology, Affiliated Hospital of Qingdao University, Qingdao, Shandong, China; c Department of Interventional Medicine, Affiliated Hospital of Qingdao University, Qingdao, Shandong, China.

**Keywords:** case report, pancreaticoduodenal artery aneurysm, pancreatitis

## Abstract

Pancreaticoduodenal artery aneurysm (PDAA) is rare and has high rupture risks. PDAA rupture has a wide range of clinical symptoms, including abdominal pain, nausea, syncope, and hemorrhagic shock, which is difficult to differentiate from other diseases.

**Patient concerns:**

A 55-year-old female patient was admitted to our hospital due to abdominal pain for 11 days.

**Diagnosis:**

Acute pancreatitis was initially diagnosed. The patient’s hemoglobin decreased compared to before admission, suggesting that active bleeding may occur. CT volume diagram and maximum intensity projection diagram show that a small aneurysm with a diameter of about 6 mm can be seen at the pancreaticoduodenal artery arch. The patient was diagnosed with a rupture and hemorrhage of the small pancreaticoduodenal aneurysm.

**Interventions:**

Interventional treatment was performed. After the microcatheter was selected for the branch of the diseased artery for angiography, the pseudoaneurysm was displayed and embolized.

**Outcomes:**

The angiography showed that the pseudoaneurysm was occluded, and the distal cavity was not redeveloped.

**Conclusion:**

The clinical manifestations of PDAA rupture were significantly correlated with the aneurysm diameter. Because of small aneurysms, the bleeding is limited around the peripancreatic and duodenal horizontal segments, accompanied by abdominal pain, vomiting, and elevated serum amylase, similar to the clinical manifestations of acute pancreatitis but accompanied by the decrease of hemoglobin. This will help us to improve our understanding of the disease, avoid misdiagnosis, and provide the basis for clinical treatment.

## 1. Introduction

Pancreatoduodenal aneurysm (PDAA) is an exceedingly rare visceral artery located between the celiac artery (CA) and the superior mesenteric artery and has a high risk of rupture.^[1]^ PDAA is affected by hemodynamic changes, and its formation is closely related to CA stenosis or occlusion.^[2,3]^ The risk of PDAA rupture is independent of its diameter, and the mortality rate after PDAA rupture is high (21–26%).^[2,4]^ Endovascular or interventional treatment is recommended at the time of diagnosis.^[4,5]^ Although the early diagnosis of the disease is essential, PDAA rupture is difficult to distinguish from other abdominal disorders.

This study reported a small aneurysm with a diameter of <1 cm. Because of small aneurysms, the bleeding is limited around the peripancreatic and duodenal horizontal segments. The clinical manifestations were similar to those of acute pancreatitis, including abdominal pain, vomiting, and elevation of serum amylase, but accompanied by a decrease of hemoglobin (Hb). This reminds clinicians to be alert to small aneurysm bleeding when encountering acute abdomen with no apparent gastrointestinal bleeding and abdominal or retroperitoneal bleeding.

## 2. Case report

**Figure 1.**
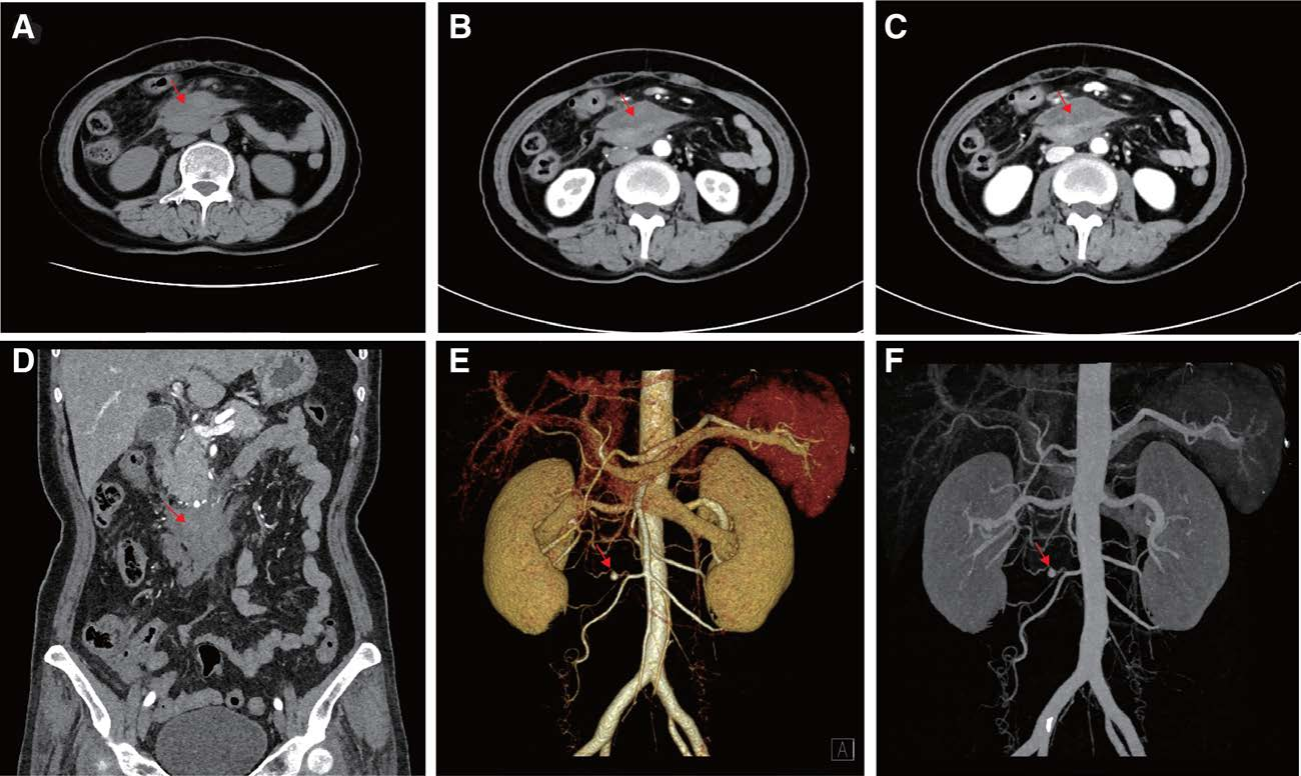
(A) nonenhanced CT shows a mass in front of the duodenum, under the head of the pancreas. The attenuation is heterogeneous, and the CT value of the patchy high-density area within the mass is 49 Hu, indicating hemorrhage. On the arterial (B) and venous (C) contrast-enhanced CT images, the mass shows heterogeneous density with no noticeable enhancement, suggesting the diagnosis of a hematoma. (D) The coronal multiplanar reformation image shows a small aneurysm above the hematoma with a diameter of 6 millimeters. The volume rendering (E) and maximum intensity projection (F) CT images show an aneurysm in the arch of the pancreaticoduodenal artery.

A 55-year-old female patient was admitted to the Affiliated Hospital of Qingdao University on February 25, 2022, due to “abdominal pain for 11 days.” On February 14, 2022, the patient developed epigastric pain after tiredness, initially paroxysmal colic, then gradually aggravated, presenting continuous total abdominal pain, accompanied by vomit of stomach contents. After vomiting, the abdominal pain did not relieve, and no hematemesis, fever, or diarrhea, so he came to our hospital for emergency treatment. She has been in good health. There is nothing special about family history. Physical examination: sad face, full abdominal tenderness, no rebound pain. White blood cell 11.09 × 109/L, neutrophil count 9.22 × 109/L, Red blood cell 3.83 × 1012/L, Hb 125g/L, Platelets, C-reactive protein 15.6, blood amylase 309U/L, upper abdominal CT showed that the head of the pancreas was plump with peripheral changes, the boundary between the head of the pancreas and the horizontal segment of the duodenum was unclear, and inflammatory changes were possible (Fig. 1A), so it was admitted to our department for treatment as “acute pancreatitis.”

**Figure 2.**
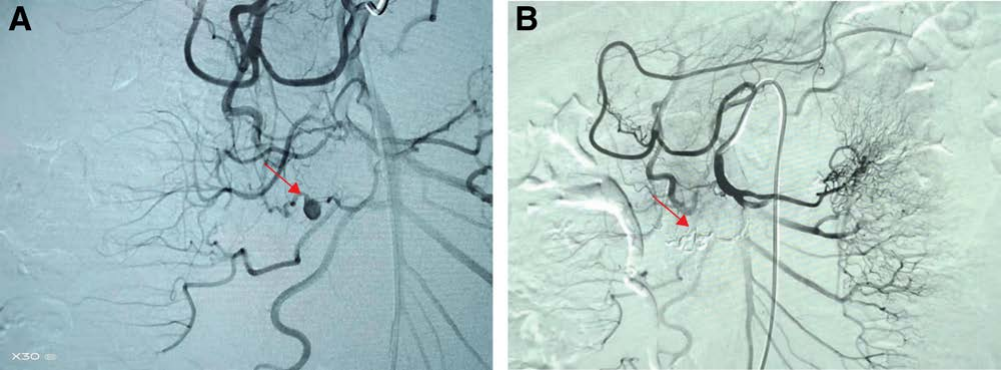
(A) Superior mesenteric artery for angiography showed a pseudoaneurysm at the pancreaticoduodenal arch. (B) The angiography showed that the pseudoaneurysm was occluded, and the distal cavity was not redeveloped. (C) CT of the upper abdomen showed what was seen after celiac artery embolization.

After admission, Grade I nursing, diet ban, PPI, somatostatin, antiinfection, and fluid resuscitation were given to reduce the degree and scope of abdominal pain and obvious tenderness in the middle and upper abdomen. White blood cell examination on February 27, 2022 7.11 × 109/L, neutrophil count 4.76 × 109/L, Red blood cell 3.54 × 1012/L, Hb 112g/L, Platelets, C-reactive protein 15.37, blood amylase 85U/L, blood glucose 6.14 mmol/L, blood calcium 2.19 mmol/L. Hemagglutination, liver function, kidney function, electrolyte, tumor marker, antinuclear antibody, ENA zymogram, and IgG4 were normal. The patient’s Hb decreased compared to before admission, suggesting that active bleeding may occur. Gastroscopy revealed swelling and punctate congestion in the descending and horizontal parts of the duodenum. The enhanced CT of the upper abdomen showed multiple masses around the head of the pancreas and the horizontal segment of the duodenum. The enhancement of the arterial phase (Fig. 1B) and venous phase (Fig. 1C) showed uneven mass density without obvious enhancement, indicating hematoma. Coronal multiplanar reconstruction image showed a small aneurysm with a diameter of 6 mm above the hematoma (Fig. 1D). CT volume diagram (Fig. 1E) and maximum intensity projection diagram (Fig. 1F) show that a small aneurysm with a diameter of about 6 mm can be seen at the pancreaticoduodenal artery arch. The patient was diagnosed with a rupture and hemorrhage of the small pancreaticoduodenal aneurysm. Interventional treatment was performed, 5 F was sent to the celiac trunk and the superior mesenteric artery for angiography. A pseudoaneurysm was seen at the pancreaticoduodenal arch (Fig. 2A). After the microcatheter was selected for the branch of the diseased artery for angiography, the pseudoaneurysm was displayed. After the microcatheter was selected for the proximal artery of the pseudoaneurysm, a mixture of tissue glue 1mL + iodized oil 1ml was used for embolization. Later, the angiography showed that the pseudoaneurysm was occluded, and the distal cavity was not redeveloped (Fig. 2B). CT of the upper abdomen showed what was seen after CA embolization (Fig. 2C). After the operation, the patient’s abdominal pain was relieved, disappeared, and was cured and discharged.

## 3. Discussion

PDAA is rarer, accounting for about 2% of visceral aneurysms, and the clinical symptoms are atypical, so they are often ignored. Most visceral aneurysms have no apparent symptoms before rupture, but abdominal pain, abdominal pulsating mass, and other symptoms may occur with the increase of the aneurysm body; Once fracture occurs, there may be abdominal and retroperitoneal bleeding, gastrointestinal bleeding, shock, etc. Without intervention, the pancreaticoduodenal aneurysm and gastroduodenal aneurysm rupture rate can reach 80% to 100%.^[6]^ In 50% to 80% of cases, PDAA is associated with occlusive arterial disease involving the opening of the main artery.^[7,8]^ The hypothesis of high dynamic collateral circulation proposed that based on celiac trunk or superior mesenteric artery occlusion, the blood flow velocity to the pancreatic artery arch increases, leading to the progressive expansion of the arterial cavity.^[2]^ Recent dynamic imaging studies confirm this effect.^[3,4,9,10]^ Other reported causes were atherosclerosis, connective tissue disease, and infection.^[11,12]^ In this case report, the patient has no definite cause.

The more common use and accuracy of enhanced imaging techniques, computed tomography, and 3-dimensional imaging have increased the diagnostic frequency of visceral aneurysm degeneration.^[13]^ The main treatment methods for visceral aneurysms are open surgery and endovascular therapy. The data show that endovascular techniques can be used as the first choice for treating ruptured visceral aneurysms, whether in elective surgery or emergency treatment.^[5,13,14]^ Barrionuevo et al^[15]^ found no statistically significant difference in mortality between open surgery and endovascular therapy through systematic review and meta-analysis. Surgeons are more likely to use intravascular therapy because of shorter hospital stays and a lower rate of cardiovascular complications, but there is a higher rate of reintervention. For PDAA with celiac trunk obstruction, surgical treatment may be a better solution, considering the possible hepatic ischemia during the endovascular embolization of the pancreaticoduodenal artery and the low expected embolization efficiency.^[16]^

Through diagnosing and treating this patient with a pancreaticoduodenal aneurysm, we reviewed the relevant literature and made a summary and reflection; CT enhanced examination and 3-dimensional reconstruction can clearly diagnose the aneurysm. In this case, 3-dimensional reconstruction can display the pancreaticoduodenal aneurysm. For patients with acute abdomen, if the condition and conditions permit, abdominal CT enhancement examination should be given priority to understand the disease better and obtain a more accurate diagnosis; For patients with a clinical diagnosis similar to acute pancreatitis, if the blood picture shows that Hb is decreased, accompanied by gastrointestinal bleeding, nontraumatic intraperitoneal or retroperitoneal bleeding, the possibility of visceral aneurysm rupture and the bleeding should be ruled out first; PDAA rupture with a smaller diameter may not show apparent gastrointestinal bleeding or intraperitoneal or retroperitoneal hematoma. The results of CT and hemogram should be combined to improve vigilance; Endovascular treatment is the first choice for unruptured visceral aneurysms to fully understand the potential risk of rupture and bleeding. The situation after rupture and hemorrhage of the visceral aneurysm is hazardous, especially the rupture of a pancreaticoduodenal aneurysm, which has a very high mortality rate. Therefore, it should be timely evaluated, and appropriate treatment should be selected according to the specific situation.

## 4. Conclusion

In summary, pancreatoduodenal aneurysms without obvious causes are rare in clinical practice. As the first choice for diagnosis, abdominal CTA is of great value. Later reconstruction technology can accurately locate the bleeding site and identify the tumor body, providing detailed information for diagnosis and treatment options. This study reported a small aneurysm with a diameter of <1 cm. Its clinical manifestations and serological indicators were similar to those of acute pancreatitis but with a decrease in Hb. This reminds clinicians to be alert to atypical presentations of arteriolar hemorrhage. This will help us to improve our understanding of the disease and avoid misdiagnosis.

## Acknowledgments

The authors would like to thank the patient and her family for providing informed consent for publication.

## Author contributions

**Data curation:** Ya Nan Yu, Cong Cong Min, Xue Li Ding.

**Investigation:** Xue Li Ding.

**Methodology:** Pei Nie, Cong Cong Min, Guo Ping Liu, Tao Mao.

**Project administration:** Yong Hong Xu.

**Resources:** Guo Ping Liu, Tao Mao.

**Supervision:** Yong Hong Xu.

**Visualization:** Tao Mao.

**Writing – original draft:** Yu Shuang Xu.

**Writing – review & editing:** Ya Nan Yu.
